# Early cognitive and emotional outcome after stroke is independent of discharge destination

**DOI:** 10.1007/s00415-020-09999-7

**Published:** 2020-06-24

**Authors:** Jos P. L. Slenders, Daan P. J. Verberne, Johanna M. A. Visser-Meily, Renske M. Van den Berg-Vos, Vincent I. H. Kwa, Caroline M. van Heugten

**Affiliations:** 1 Department of Neurology, OLVG, Amsterdam, The Netherlands; 2 Department of Psychiatry and Neuropsychology, Maastricht University, Maastricht, The Netherlands; 3 Department of Rehabilitation, Physical Therapy Science and Sports, UMC Utrecht Brain Center, University Medical Center Utrecht, Utrecht, The Netherlands; 4 Center of Excellence for Rehabilitation Medicine, UMC Utrecht Brain Center, University Medical Center Utrecht, and De Hoogstraat Rehabilitation, Utrecht, The Netherlands; 5 Department of Neurology, Amsterdam UMC, Amsterdam, The Netherlands; 6 Department of Neuropsychology and Psychopharmacology, Maastricht University, PO Box 616, 6200 MD Maastricht, The Netherlands

**Keywords:** Cerebrovascular disease/stroke, Cognition, Anxiety and depression, Participation

## Abstract

**Background and purpose:**

Cognitive and emotional problems occur frequently after stroke. Patients with minor stroke are more likely to be discharged home. This paper compares early cognitive and emotional outcomes in patients discharged home after stroke versus patients discharged to inpatient rehabilitation, and examines the effect of cognitive and emotional outcomes on long-term participation.

**Methods:**

In this multicenter prospective cohort study, patients with stroke were assessed at two months with the Hospital Anxiety and Depression Scale (HADS), the Checklist for Cognitive and Emotional Consequences following Stroke (CLCE-24) and the Montreal Cognitive Assessment (MoCA). One year post stroke, participation was assessed with the Restriction subscale of the Utrecht Scale for Evaluation of Rehabilitation—Participation (USER-P Restriction).

**Results:**

The study included 332 patients. Two months post stroke, anxiety and cognitive problems were equally prevalent among patients discharged home (*n* = 243; 73%) and patients discharged to inpatient rehabilitation (*n* = 89; 27%) (HADS-A = 4.8 ± 3.9 versus 4.6 ± 4.0, *p* = 0.747; MoCA < 26: 66.7% versus 70.8%, *p* = 0.477; CLCE-cognition = 3.0 ± 2.9 versus 3.3 ± 2.8, *p* = 0.499). Depressive symptoms were less severe in patients discharged home (HADS-D = 4.3 ± 3.9 versus 5.5 ± 3.8, *p* = 0.010). In patients discharged home, cognitive complaints were predictive of long-term participation (*B* = − 2.03; 95% CI − 3.15, − 0.90), while cognitive or emotional outcomes were not predictive in patients discharged to inpatient rehabilitation.

**Conclusions:**

Cognitive and emotional problems at two months post stroke were comparable between patients discharged home and those discharged to inpatient rehabilitation. For patients discharged home, cognitive complaints were predictive of long-term participation.

## Introduction

Global incidence and prevalence of stroke are both high, leaving many patients disabled [1–4]. Discharge destination after hospital admission is largely determined by functional dependence and the level of physical disability [5]. Patients who have suffered a minor stroke are more likely to be considered functionally independent and will most likely be discharged home directly, whereas patients with a major stroke are more likely to be discharged to inpatient rehabilitation [6]. After inpatient rehabilitation, the vast majority of these patients will also be discharged home. Recent developments in the acute care for ischemic stroke show decreases in the level of physical disability [7, 8]. As a result, the number of so-called ‘walking and talking’ patients (i.e. patients with relatively good neurological recovery) is increasing and more patients are being discharged home directly [9, 10].

Besides physical disability, cognitive and emotional problems are frequent after stroke, among both patients with major and minor strokes [11, 12]. These problems can negatively affect participation (for example in household activities, return to work or social activities), which is an important outcome of stroke care and an essential goal in stroke rehabilitation [13]. Screening and treatment for cognitive and emotional problems is an integral part of inpatient rehabilitation, whereas this type of treatment is possibly less commonly offered to patients who are discharged home [14]. As these problems are less obvious than physical disability, recognition and effective follow-up care might be lacking in patients recovering at home, even though they may also need follow-up care [15, 16]. To the best of our knowledge, no direct comparison has been made of cognitive and emotional outcomes in the early phase after stroke between patients discharged home and those discharged to inpatient rehabilitation.

This study aimed to compare early cognitive and emotional problems after stroke in patients discharged home versus patients discharged to inpatient rehabilitation. Additionally, the effects of early cognitive and emotional outcomes on participation at one year post stroke were examined in both groups, while controlling for demographic and stroke characteristics.

## Methods

### Design and procedure

The current study concerns secondary analyses of the Restore4stroke Cohort, a multicenter prospective longitudinal cohort study conducted in six general hospitals in the Netherlands [17]. Patients were included between March 2011 and March 2013.

The Restore4stroke study consisted of five assessments from stroke onset up to 24 months post stroke. The current study used data from within the first week (T1) and two months (T2) and one year (T3) post stroke. When eligibility criteria were met and written informed consent had been obtained, research nurses extracted demographic and medical information from the medical charts after one week. The assessment, including a cognitive assessment at two months, was performed by a trained research assistant. The assessment at one year consisted solely of questionnaires and was completed by the patient on paper or online. The protocol has been described in more detail elsewhere [17].

### Participants

Patients with a clinical diagnosis of stroke (either ischemic or hemorrhagic) in the past seven days, as confirmed by a neurologist, were considered eligible. Patients were excluded if one of the following was present: (1) comorbidity interfering with the study outcomes, (2) dependence in activities of daily living (ADL) before the stroke, as defined by a Barthel Index (BI) score of 17 or lower, (3) insufficient command of the Dutch language, based on clinical judgement, and (4) cognitive decline as defined by a score of one or higher on the Heteroanamnesis List Cognition before their stroke [18].

### Measures

#### Demographics and stroke characteristics

Demographic information included sex, age, marital status, discharge destination and level of education according to the Dutch classification system developed by Verhage [19]. Stroke characteristics included stroke severity, measured by the National Institutes of Health Stroke Scale (NIHSS) [20]. Functional dependence in ADL was measured by the BI, with a total score ranging from zero to 20; higher scores are indicative of greater independence in ADL [21]. Patients discharged home were defined as those being discharged home directly after their hospital stay and still living at home after two months. All others were discharged to inpatient rehabilitation (including geriatric rehabilitation) with the aim of living at home again within three to six months after the diagnosis was made.

#### Cognitive functioning

The Montreal Cognitive Assessment (MoCA) was used to screen for the presence of cognitive disorders. The MoCA consists of ten items with a total score ranging from 0 to 30. A higher score reflects a better performance. A score < 26 was regarded as cognitive impairment [22]. The MoCA was completed by a trained research assistant.

#### Cognitive and emotional complaints

The Checklist for Cognitive and Emotional Consequences following Stroke (CLCE-24) is a patient-reported outcome measure. It was used to assess the number of cognitive and emotional complaints in daily life and was completed by a trained research assistant. The cognitive domain (CLCE-24 cognition) consists of 13 items (ranging from 0 to 13) and the emotional domain (CLCE-24 emotion) of nine items (ranging from 0 to 9). The instrument includes two blank items in case other problems are present which are not mentioned in the list of common consequences. All items are scored as ‘absent (0)’ or ‘present (1)’ (‘present’ or ‘doubtful’) [23].

#### Anxiety and depression

The Hospital Anxiety and Depression Scale (HADS) was used to assess the severity of symptoms of anxiety and depression. The HADS includes seven items for both the anxiety (HADS-A) and depression (HADS-D) subdomains, resulting in 14 items in total. Each item is rated on a four-point scale (0–3) and a higher score reflects more severe symptoms. Both subdomain scores range from 0 to 21. ‘No symptoms’ was defined as a score < 8 for each subdomain and ‘mild to severe symptoms of depression or anxiety’ was defined as a score ≥ 8 [24, 25].

#### Participation

The Restriction subscale of the Utrecht Scale for Evaluation of Rehabilitation-Participation (USER-P Restriction) was used to measure if subdomains of participation (e.g. ‘work’ or ‘relationship’) could be performed ‘independently without difficulty’, ‘with difficulty’, ‘with assistance’ or ‘cannot be performed’. The USER-P Restriction consists of 11 items and compares the current situation with the situation before the stroke for each item. The sum of the items is converted to a 0–100 scale, with a higher score reflecting less restrictions [26]. The USER-P Restriction has previously demonstrated satisfactory validity and reliability, and it has excellent responsiveness in patients after stroke [27–29].

### Statistical analyses

All patients who completed the MoCA, CLCE-24 and HADS at two months were included in this analysis. Patients were divided into those discharged home and those discharged to inpatient rehabilitation.

Demographics and stroke-related information were recorded. Marital status was recorded as being or not being in a relationship. Type of stroke was recorded as ischemic or hemorrhagic stroke. Educational level was dichotomized into ‘low’ (≤ 5) versus ‘high’ (≥ 6; i.e. completed higher professional education or university) based on the Dutch classification system developed by Verhage [19]. Selection bias analyses were performed in which included patients and excluded patients (with missing data at two months) were compared on demographic and stroke-related information. Independent samples *t* tests were performed to examine the differences between the two groups at two months on the MoCA, CLCE-24 cognition, CLCE-24 emotion, HADS-A and HADS-D. Pearson chi-square statistics were used to examine differences in percentages of patients with a HADS-A and HADS-D above the cutoff points and MoCA below the cutoff point.

Restrictions in participation one year after stroke, measured by the USER-P Restriction, were dichotomized into ‘no restrictions’ (‘independently without difficulty’) and ‘restrictions’ (‘with difficulty’, ‘with assistance’ or ‘cannot be performed’) and were analyzed using descriptive statistics.

Next, the association of the predictors of interest, viz. the MoCA, CLCE-24 cognition, CLCE-24 emotion, HADS-A and HADS-D scores, with USER-P Restriction at one year were examined using univariable linear regression. A multivariable model was used to analyze significantly associated predictors of interest. Besides the significantly associated predictors of interest, the multivariable model was adjusted for the following pre-specified demographic and stroke-related covariates: sex, age at stroke, marital status, educational level, type of stroke, stroke severity as measured with the NIHSS, functional dependence as measured with the BI, and length of hospital stay in days. The univariable associations and multivariable models were performed separately for the patients discharged home and those discharged to inpatient rehabilitation.

The assumptions of linearity, independent errors, homoscedasticity, and normally distributed errors were checked. A *p* value of 0.05 was used to determine statistical significance. IBM SPSS version 25.0 was used for analyses.

## Results

### Characteristics

The Restore4stroke Cohort study included 395 patients. Sixty-three patients were excluded because MoCA, CLCE-24 or HADS data at two months were missing. A total of 332 patients with complete data were included in the current analysis. Baseline data of these patients are presented in Table 1. Excluded patients (*n* = 63) had a significantly higher NIHSS score (4.2 ± 4.2; *p* = 0.004) and a significantly lower BI (15.0 ± 4.4; *p* = 0.002) than the group of included patients.

**Table 1:** Baseline characteristics of the total group and specified for discharge destination

	Total group (*n* = 332)	Discharged home (*n* = 243)	Discharged to inpatient rehabilitation (*n* = 89)	*p* value
Sex (% male)	64.5	66.7	58.4	0.165
Age in years (mean ± SD)	66.7 ± 12.3	65.2 ± 11.8	70.7 ± 12.9	< 0.001
Marital status (% in relationship)	68.7	76.1	48.3	< 0.001
High education level (%)	26.2	28.0	21.3	0.223
Ischemic stroke (%)	93.1	94.2	89.9	0.256
Location of stroke				0.077
Left hemisphere (%)	40.5	43.8	31.8	
Right hemisphere (%)	42.4	38.3	53.4	
Vertebrobasilar (%)	16.1	17.9	14.8	
First stroke (%)	87.0	86.0	89.9	0.722
NIHSS (mean ± SD)	2.5 ± 2.9	1.7 ± 2.2	4.6 ± 3.7	< 0.001
BI (mean ± SD)	17.2 ± 4.4	18.8 ± 2.5	12.7 ± 5.3	< 0.001
Length of hospital stay in days (mean ± SD)	8.5 ± 6.4	6.3 ± 3.7	14.5 ± 8.1	< 0.001

*SD* standard deviation, *NIHSS* National Institutes of Health Stroke Scale, *BI* Barthel Index

Of the total group, 243 (73%) were discharged home and 89 (27%) were discharged to inpatient rehabilitation. The group of patients discharged home was significantly younger, had a lower NIHSS score, a higher BI score, and a shorter hospital stay, and was significantly more likely to be in a relationship (*p* < 0.05) (Table 1). Of the patients who were discharged to inpatient rehabilitation, 92% were living at home after one year; the remaining 8% were living at inpatient rehabilitation at one year.

### Comparison of cognitive and emotional consequences at two months after stroke

The results of the MoCA, CLCE-24 cognition, CLCE-24 emotion and HADS-A assessments at two months did not differ significantly between the patients discharged home and those discharged to inpatient rehabilitation (Table 2). Patients discharged to inpatient rehabilitation scored significantly higher on the HADS-D than those discharged home (5.5 ± 3.8 versus 4.3 ± 3.9). The proportion of cognitively impaired patients with a score < 26 on the MoCA did not differ significantly between the two groups. The proportions of patients with above-cutoff scores on the HADS-A and HADS-D did not differ significantly between groups either.

**Table 2:** Cognitive and emotional outcomes at two months in patients discharged home and patients discharged to inpatient rehabilitation group

	Discharged home (*n* = 243)	Discharged to inpatient rehabilitation (*n* = 89)	*p* value
MoCA (mean ± SD)	23.8 ± 3.8	22.9 ± 4.1	0.067
% MoCA < 26 cutoff	66.7	70.8	0.477
CLCE-24 cognition (mean ± SD)	3.0 ± 2.9	3.3 ± 2.8	0.499
CLCE-24 emotion (mean ± SD)	2.8 ± 1.9	3.0 ± 1.8	0.451
HADS-D (mean ± SD)	4.3 ± 3.9	5.5 ± 3.8	0.010
HADS-A (mean ± SD)	4.8 ± 3.9	4.6 ± 4.0	0.747
%HADS-D ≥ 8 cutoff	19.3	25.8	0.198
% HADS-A ≥ 8 cutoff	19.3	21.3	0.685

*MoCA* Montreal Cognitive Assessment, *CLCE-24* Checklist for Cognitive and Emotional Consequences following Stroke, *HADS* Hospital Anxiety and Depression Scale

### Restrictions in participation one year after stroke

Table 3 displays the level of patient-reported restrictions in participation at one year for each of the subdomains. Overall, patients discharged to inpatient rehabilitation experienced more restrictions in participation after one year. The four most affected domains in both groups were paid/unpaid work or education, sports and physical exercise, household activities and day trips.

**Table 3:** Restrictions in participation after one year for the total group

Items	Discharged home (*n* = 243)		Discharged to inpatient rehabilitation (*n* = 89)	
	*n*	% of patients experiencing restrictions	*n*	% of patients experiencing restrictions
Paid/unpaid work/education	90	46.7	18	83.3
Household activities	199	42.2	68	80.9
Outside activities	202	33.2	71	59.2
Sports/physical exercise	188	47.9	62	75.8
Going out	161	34.8	54	68.5
Day trips	180	40.0	65	80.0
Leisure activities	193	22.8	74	39.2
Relationship with partner	163	30.1	35	57.1
Visiting friends	202	30.7	68	58.8
Receiving visitors	205	18.5	69	30.4
Contact online or by telephone	200	17.0	72	22.2

### Predictive value of cognitive and emotional problems on participation in patients discharged home

Univariable linear regression for patients discharged home showed that the MoCA, CLCE-24 cognition, CLCE-24 emotion, HADS-A and HADS-D scores were significantly associated with USER-P Restriction scores (for participation) at one year (*p* < 0.05) (Table 4). Multivariable linear regression, adjusted for sex, age at stroke, marital status, educational level, type of stroke, stroke severity as measured with the NIHSS, functional dependence as measured with the BI, and length of hospital stay in days, showed that only CLCE-24 cognition scores and the covariate of age had a significant negative effect on participation at one year. The MoCA, CLCE-24 emotion, HADS-A and HADS-D scores were not significantly predictive of participation at one year.

**Table 4:** Linear regression: effects of emotional and cognitive problems at two months on participation at one year after stroke

Predictors at two months post stroke	USER-P Restrictions at one year post stroke					
	Univariable analyses			Multivariable analysis^a^		
	B (95% CI)	SE	*p* value	B (95% CI)	SE	*p* value
Patients discharged home directly						
MoCA	1.394 (0.712 to 2.076)	0.346	< 0.001	0.555 (− 0.144 to 1.255)	0.355	0.119
CLCE-24 cognition	− 3.017 (− 3.787 to − 2.248)	0.390	< 0.001	− 2.025 (− 3.153 to − 0.897)	0.572	< 0.001
CLCE-24 emotion	− 3.508 (− 4.722 to − 2.294)	0.616	< 0.001	− 0.014 (− 1.653 to 1.625)	0.831	0.987
HADS-D	− 2.048 (− 2.611 to − 1.485)	0.285	< 0.001	− 0.743 (− 1.681 to 0.195)	0.476	0.120
HADS-A	− 1.926 (− 1.926 to − 1.324)	0.305	< 0.001	− 0.527 (− 1.433 to 0.379)	0.459	0.252
Patients discharged to inpatient rehabilitation						
MoCA	0.410 (− 0.688 to 1.508)	0.551	0.459	NA	–	–
CLCE-24 cognition	0.104 (− 1.547 to 1.755)	0.829	0.901	NA	–	–
CLCE-24 emotion	− 0.359 (− 2.904 to 2.185)	1.278	0.779	NA	–	–
HADS-D	− 0.158 (− 1.312 to 0.995)	0.579	0.785	NA	–	–
HADS-A	− 0.017 (− 1.079 to 1.113)	0.550	0.976	NA	–	–

*CI* confidence interval, *CLCE-24* Checklist for Cognitive and Emotional Consequences following Stroke, *HADS* Hospital Anxiety and Depression Scale, *MoCA* Montreal Cognitive Assessment, *NA* not applicable, *SE* standard error

^a^Adjusted for sex, age at stroke, marital status, educational level, type of stroke, NIHSS, BI and length of hospital stay in days

### Predictive value of cognitive and emotional problems on participation in patients discharged to inpatient rehabilitation

In the inpatient rehabilitation group, none of the predictors of interest proved to be significantly predictive of USER-P Restriction scores in univariable linear regression (Table 4). As a consequence, no multivariable analysis was performed for the inpatient rehabilitation group.

## Discussion

Remarkably, this study showed that cognitive problems and symptoms of anxiety and depression are comparable at two months after stroke for patients discharged home and patients discharged to inpatient rehabilitation. We further found that, in patients discharged home, cognitive complaints at two months after stroke were predictive of participation restrictions at one year after stroke, when adjusted for demographic and stroke characteristics, including stroke severity. In patients discharged to inpatient rehabilitation, none of the cognitive or emotional scores were predictive of participation at one year.

After stroke, whether a patient is discharged home or to inpatient rehabilitation predominantly depends on functional dependence: the ability of a person to carry out daily activities in a safe and autonomous manner [6]. Functional dependence, in turn, is highly dependent on the physical outcome after stroke [30]. However, the results presented in this article show that patients with relatively good physical outcome who are discharged home experienced just as many cognitive and emotional problems as patients discharged to inpatient rehabilitation. Apparently, cognitive and emotional outcomes were not closely related to stroke severity as defined by physical disability. This gives rise to the question whether stroke severity should be defined predominantly by physical disability.

The high prevalence of cognitive and emotional problems was remarkable. This high prevalence among all our stroke patients, whether with or without substantial physical disability, might be due to damage to the complex underlying neural network. Since optimal cognitive and emotional functioning is supported by an extensive neural network, any stroke is likely to influence this network and its functions [31–33].

Additionally, the current study shows that cognitive complaints were prognostic of long-term participation restrictions in patients discharged home, which is in line with a recently published systematic review [34]. However, the results of individual studies in this review were mixed, and the authors stated that this variation could be explained by the limited predictive value of general screening tools. This might explain why the MoCA (as a screening tool) was not predictive in the current study, whereas the patient-reported CLCE-24 cognition was. Apparently, cognitive complaints that do not interfere with functional dependence, do interfere with long-term participation. This sounds reasonable, since participation across multiple domains is highly complex and is associated with multitasking and time pressure in many instances. As such, optimal participation requires great effort in terms of cognitive and emotional functioning. For patients discharged to inpatient rehabilitation, none of the cognitive or emotional problems were associated with long-term participation. As an implication for clinical practice, the results of this study underline the recommendation to actively screen for cognitive and emotional problems in an early phase after stroke in all patients after stroke and for any post-discharge setting [35]. Early detection and management for cognitive and emotional problems has proven its efficacy in terms of clinical and cost-effectiveness in patients after cardiac arrest [36, 37]. Moreover, patients with cognitive problems can benefit from cognitive rehabilitation by learning compensational strategies, and patients with emotional problems can be treated by psychological interventions, such as cognitive behavioral therapy, and/or pharmacotherapy. Moreover, rehabilitation care (whether inpatient or outpatient) can improve participation, including return to work [38–40].

The strengths of this study are its prospective nature, as well as the multicenter design. In addition, it included a large group of patients who completed long-term follow-up measurements. Cognitive and emotional outcomes were measured with validated patient-reported and objective screening instruments. Since participation is one of the main priorities in stroke and rehabilitation care, understanding its predictors is highly relevant for clinical practice. A limitation of this study is that the majority of the study sample had suffered a minor stroke and the number of patients with hemorrhagic stroke was rather small, which limits the generalizability of findings to the whole stroke population. Second, the group discharged to inpatient rehabilitation was relatively small which reduced the statistical power of the analyses in this group.

## Conclusion

In conclusion, a surprising finding of this study was that cognitive and emotional problems were equally prevalent and severe among patients discharged home directly and those discharged to inpatient rehabilitation after stroke. Multivariable regression analyses revealed that cognitive complaints were predictive of long-term participation restrictions in patients discharged home. These findings underline the guideline recommendations on screening and treatment for cognitive and emotional problems, including for patients with minor stroke who are discharged home.

## Data Availability

The data that support the findings of this study are available from the corresponding author upon reasonable request.
